# Effects of crystalloid and colloid priming strategies for cardiopulmonary bypass on colloid oncotic pressure and haemostasis: a meta-analysis

**DOI:** 10.1093/icvts/ivac127

**Published:** 2022-05-05

**Authors:** Anne Maria Beukers, Jamy Adriana Catharina de Ruijter, Stephan Alexander Loer, Alexander Vonk, Carolien Suzanna Enna Bulte

**Affiliations:** 1 Amsterdam UMC Location Vrije Universiteit Amsterdam, Department of Anaesthesiology, Amsterdam, Netherlands; 2 Amsterdam UMC Location University of Amsterdam, Department of Cardiothoracic Surgery, Amsterdam, Netherlands

**Keywords:** Colloid oncotic pressure, Prime fluid, Cardiopulmonary bypass, Cardiac surgery

## Abstract

**OBJECTIVES:**

Colloid oncotic pressure (COP) is an important factor in cardiac surgery, owing to its role in haemodilution. The effect of cardiopulmonary bypass prime fluids on the COP is unknown. In this study, the effect of crystalloid and colloid prime fluids, with or without retrograde autologous priming (RAP), on the COP during elective cardiac surgery was evaluated.

**METHODS:**

Randomized controlled trials and prospective clinical trials comparing crystalloid and colloid priming fluids or with RAP were selected. The primary outcome was the COP; secondary outcomes were fluid balance, fluid requirements, weight gain, blood loss, platelet count and transfusion requirements.

**RESULTS:**

From 1582 records, 29 eligible studies were identified. COPs were comparable between gelofusine and hydroxyethyl starch (HES) during bypass [mean difference (MD): 0.69; 95% confidence interval (CI): −2.05, 3.43; *P *=* *0.621], after bypass (MD: −0.11; 95% CI: −2.54, 2.32; *P *=* *0.930) and postoperative (MD: −0.61; 95% CI: −1.60, 0.38; *P *=* *0.228). Fluid balance was lower with HES than with crystalloids. RAP reduced transfusion requirements compared with crystalloids. Blood loss was comparable between groups.

**CONCLUSIONS:**

COPs did not differ between crystalloids and colloids. As a result of increased transcapillary fluid movement, fluid balance was lower with HES than with crystalloids. Haematocrit and transfusion requirements were comparable between groups. However, the latter was lower when RAP was applied to crystalloid priming compared with crystalloids alone. Finally, no differences in blood loss were observed between the groups.

**Figure:**
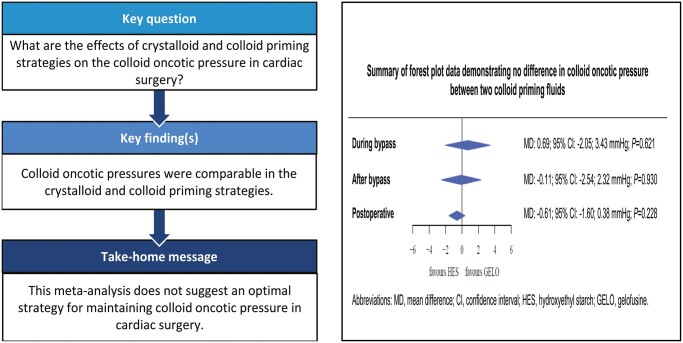

## INTRODUCTION

Colloid oncotic pressure (COP), determined by all plasma proteins in the intra- and extravascular compartments, plays a key role in transcapillary fluid movement. A decreased COP increases transcapillary fluid movement, which leads to tissue oedema and, combined with haemodilution, may compromise peripheral tissues oxygenation and end-organ perfusion [1]. In cardiac surgery, the extent of haemodilution is partly determined by the type and amount of cardiopulmonary bypass (CPB) priming fluids. However, the optimal type and amount of CPB priming fluids for cardiac surgery remain unknown.

Traditionally, crystalloids are used as priming fluid for CPB, leading to increased urine output compared to colloids [2]. However, unbalanced fluids may cause a metabolic acidic state at the onset of CPB, because of its low strong ion difference [3]. Moreover, crystalloids can increase fluid extravasation, partly explained by an osmotic effect [4]. In contrast, priming with colloids has several advantages. Colloids containing human albumin are associated with increased COP compared to crystalloids, resulting in reduced fluid requirements during cardiac surgery with CPB [5]. Moreover, human albumin has 2 beneficial properties. First, it protects the endothelial glycocalyx by preferentially binding to the glycocalyx, generating an endothelial surface layer [6]. Second, albumin influences haemostasis during cardiac surgery by preserving platelet count [5]. Despite these potential advantages of human albumin as a priming fluid, its cost and risk of potentially severe anaphylactic reactions limit its use [7]. In addition, there is conflicting evidence that synthetic colloids such as hydroxyethyl starch (HES) or gelatine/gelofusine may be beneficial as priming fluids for CPB. Generally, colloid fluid loading increases cardiac index more than saline in postoperative cardiac surgery patients, as a result of increased plasma volume due to preserved COP and reduced fluid extravasation with colloids [8]. However, artificial colloids may cause deleterious effects in terms of blood loss and platelet aggregation in the cardiac surgery setting [9–11].

Another method to reduce haemodilution during CPB priming is retrograde autologous priming (RAP), which uses the patient’s own blood for CPB priming, thereby reducing initial priming volume. The oncotic equilibrium after bypass initiation is re-established more rapidly with priming volume reduction and attenuates the hyperdynamic response after cardiac surgery [12]. Moreover, smaller priming volumes with RAP reduce fluid and transfusion requirements compared to non-RAP [12, 13].

Taken together, the optimal type and amount of CPB priming fluids and their effects on the COP during cardiac surgery are unknown. Therefore, we aimed to evaluate the effects of crystalloid and colloid priming solutions or with RAP on COP, haemodilution, transcapillary fluid movement, haemostasis and blood loss during elective cardiac surgery to gain more insight into an optimal CPB priming strategy.

## METHODS

This systematic review conformed to the reporting standards according to the Preferred Reporting Items for Systematic Reviews and Meta-Analyses guidelines [14]. The study protocol was registered online at the International Prospective Register of Systematic Reviews (registration number: CRD42021225480).

### Search strategy

A comprehensive search was conducted using Medline (PubMed) and Embase (1990). Google Scholar was used to find full-text articles when PubMed and Embase had missing links. The search strategy used is presented in Supplementary Material, Table S1. Ethical approval was not requested because all data were extracted from the original published reports.

### Study selection

**Figure 1:**
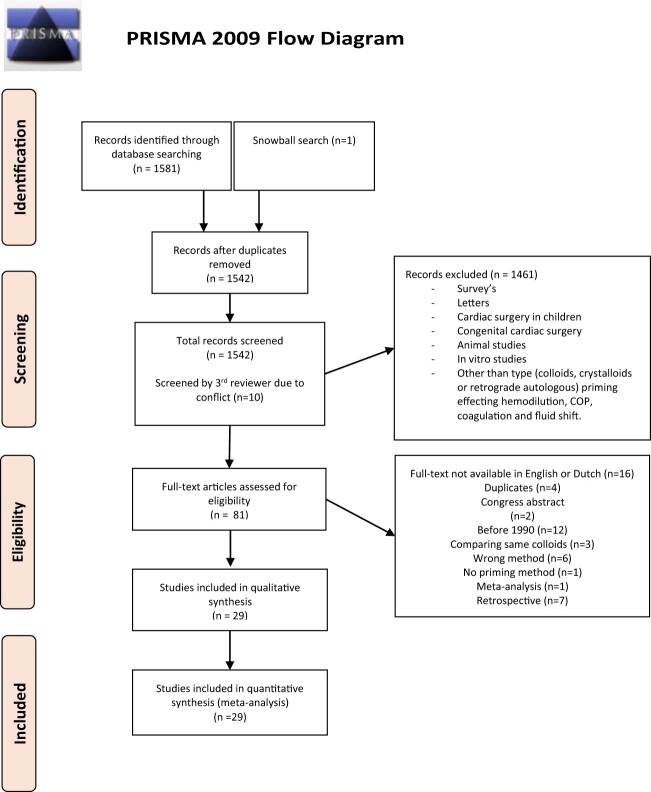
Preferred Reporting Items for Systematic Reviews and Meta-Analyses flow diagram.

Only published randomized controlled trials (RCTs) and prospective clinical trials comparing colloids (human albumin, HES or gelofusine) for CPB priming with any type of crystalloid, colloid or RAP were selected. The search was limited by age (only adult patients), language (English or Dutch), publication date (articles published after 1990) and type of subject (human). Trials by Boldt *et al.* were excluded in light of public disclosures indicating scientific misconduct by these investigators [15]. The records were entered into a database (Rayyan Qatar Computing Research Institute). Screening was independently performed by 2 reviewers (Anne Maria Beukers and Jamy Adriana Catharina de Ruijter). Initial screening for primary and secondary outcomes was based on titles and abstract, followed by full-text screening of the eligible articles for final inclusion. Duplicates were identified and removed using Rayyan. Discrepancies were resolved by a third independent reviewer (Carolien Suzanna Enna Bulte). A Preferred Reporting Items for Systematic Reviews and Meta-Analyses flow diagram (Fig. 1) was constructed to summarize the study selection process. The screening results were organized in EndNote (version X9.1).

### Data extraction

For data extraction, an electronic case report form in Castor (EDC, 2020) was used to collect the article and patient characteristics and objectives. Data were extracted by 1 reviewer (Anne Maria Beukers) and confirmed by another (Jamy Adriana Catharina de Ruijter) using *R* (version 3.6.1) for final analysis.

### Clinical outcomes

Primary outcome was COP. Secondary outcomes included fluid balance, fluid requirements, weight gain, haematocrit level, blood loss, platelet count and transfusion [packed red blood cell (PRBC) and platelet] requirements. Patient characteristics included age, sex, body mass index, body surface area, smoking status, comorbidities (diabetes mellitus and chronic obstructive pulmonary disease), European System for Cardiac Operative Risk Evaluation, CPB time and aortic cross-clamping time.

### Risk of bias

The risk of bias was determined independently by 2 reviewers (Anne Maria Beukers and Jamy Adriana Catharina de Ruijter) using the Cochrane Handbook Risk of Bias tool [16] for RCTs and the ROBINS-I tool for observational trials. Adequacy of randomization, concealment, blinding and outcome data completeness, selective outcome assessment and other possible sources of bias were determined (provided in Supplementary Material, Appendix 1).

### Statistical analysis

Statistical analysis was performed using *R* (version 3.6.1), with the package ‘meta’. All studies were tabulated with respect to their characteristics. The model used for meta-analysis was based on the degree of heterogeneity in our data. Heterogeneity was quantified using *I*^2^ statistics and was tested using Cochran’s *X*^2^ test. The results were based on a fixed-effects approach, unless the heterogeneity was significantly high (*I*^2^ > 50%), and then a random-effects approach was used. A predefined group analysis was conducted to explore the effects between crystalloids and colloids, between colloids and colloids and between crystalloids and crystalloids with RAP in patients undergoing cardiac surgery with CPB. Forest plots were created to demonstrate individual study effects and calculate pooled summary estimates. For continuous outcome variables, means with standard deviations and 95% confidence intervals (CIs) were used. Statistical significance was set at *P *<* *0.05.

## RESULTS

### Study selection and characteristics

The database search and snowball search yielded 1582 records; after duplicates removal, 1542 records were screened, of which 81 full-text articles were examined for eligibility. Finally, 29 studies were included in the meta-analysis. Twenty-six studies were RCTs, and 3 studies were observational trials. The baseline characteristics are presented in Supplementary Material, Table S2 and a summary of included studies is presented in Supplementary Material, Table S3.

### Risk of bias

Approximately 77% of the RCTs reported randomization, and only 27% reported allocation concealment. Proper blinding of participants or personnel was achieved in 46% of the trials. Attrition bias and reporting bias were considered low because most studies reported the reason for dropout or missing results (85%) and prespecified outcomes (92%), respectively. Bias for classification of interventions, deviation from intended interventions, outcomes measurement bias and selection of reported results were considered low in all observational studies. A critical risk of bias for confounding was found in 1 study and selection bias in 2 studies. Finally, a moderate risk of bias for missing data was identified in 1 study. The assessment of risk of bias is summarized in Supplementary Material, Appendix 1 and Supplementary Material, Fig. S1 and S2.

### Colloid oncotic pressure

#### Colloids versus crystalloids

Four studies reported COPs between crystalloid and colloid fluids for CPB priming [17–20]. When comparing albumin with crystalloid priming fluid, 2 studies reported COPs during and after CPB [17, 18]. Yet, these studies could not be pooled, due to different extent of data. Nevertheless, COP decreased more with crystalloids (9.0 vs 18.4 mmHg) compared with albumin (15.2 vs 19.3 mmHg) after the onset of CPB (*P *<* *0.001) and remained lower during the first 24 h after surgery (*P *<* *0.05) [18]. However, in another study, no differences in COP were found between albumin and crystalloids after surgery (1, 6 and 24 h) compared with the baseline value, despite comparable priming volumes [17]. In an RCT comparing 500 ml lactated Ringer’s (LR) priming fluid and 1000 ml gelofusine with 1500 ml LR, COP during CPB decreased with crystalloids compared with gelofusine (delta [Δ] COP: 8.5 [1.5] and 1.5 [2.9] mmHg, respectively, *P *=* *0.0001) [19]. Similarly, COP decreased from the onset of CPB until the end of bypass with crystalloids (1100 ml LR) when compared with HES (1100 ml) (*P *<* *0.05) [20]. Postoperative COPs returned to baseline values in both groups, although they remained significantly different (*P *<* *0.05). Importantly, a high molecular weight (200/0.5) 6% HES was used in this study [20]. It was shown that an increased ΔCOP during bypass was correlated with a higher fluid balance during bypass (crystalloids versus gelofusine: *r*^2^ = 0.41, *P *=* *0.002); more fluids were required during surgery when crystalloid priming fluids were used compared with gelofusine (*P *=* *0.03) [19].

#### Colloids versus colloids

**Figure 2:**
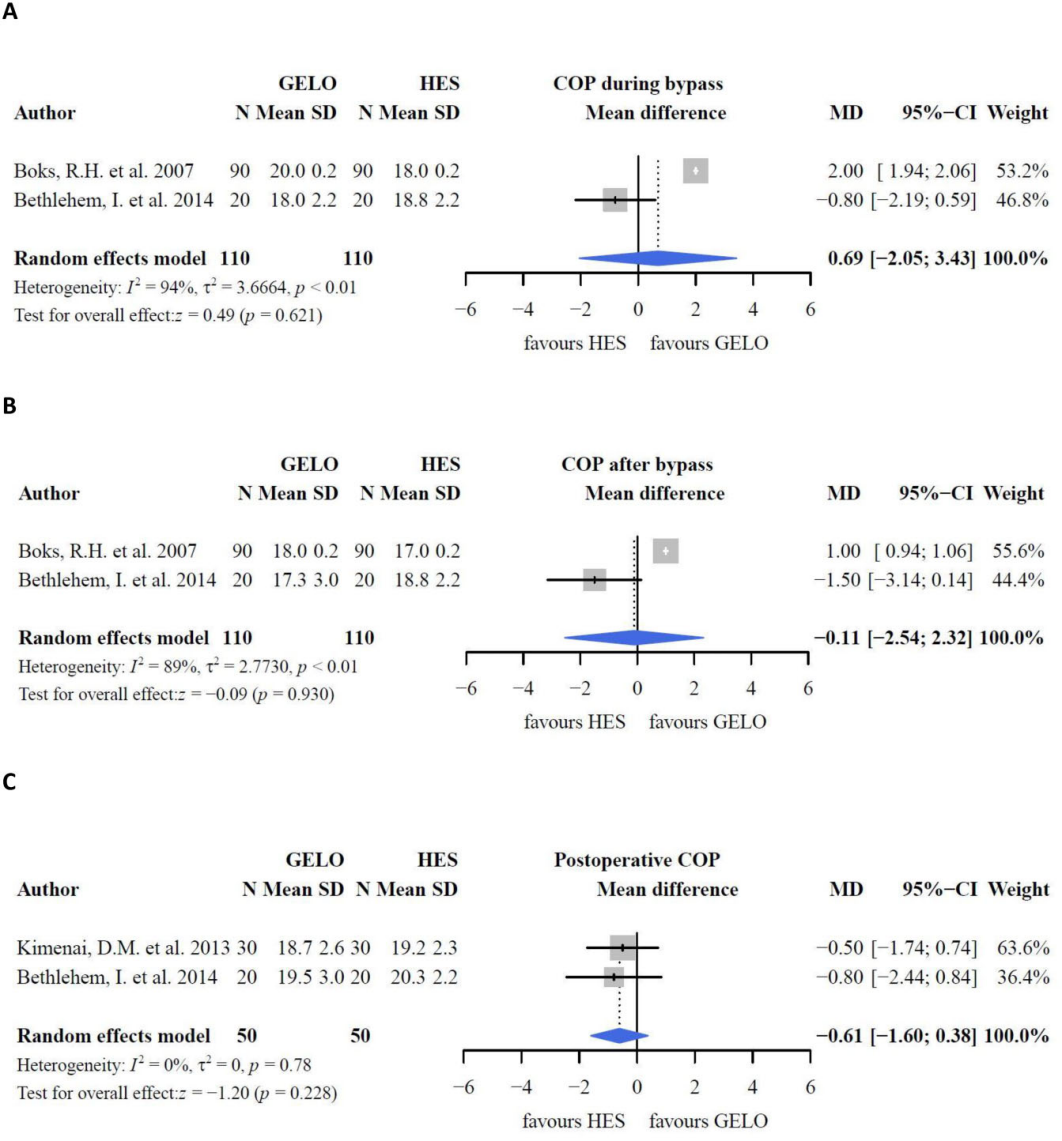
Colloid oncotic pressure.

Three studies compared perioperative COP values between HES and gelofusine [21–23]. There were no differences in baseline characteristics among the studies, except for the CPB and aortic cross-clamping times, which were longer in the HES group than in the gelofusine group (Supplementary Material, Table S2). COP during bypass decreased with HES compared with gelofusine prime fluid (*P *<* *0.05) [21]. However, in this meta-analysis, differences between HES and gelofusine as prime fluid during bypass and the postoperative period were not significant (Fig. 2). The 2 studies comparing albumin with gelofusine were not pooled because of different extent of data (medians with interquartile ranges versus means with standard deviations) [24, 25]. There were no differences in COP between the groups [25]. COP was not measured in studies comparing HES with albumin.

#### Retrograde autologous priming

Three studies compared crystalloids with a combination of crystalloids and RAP during CPB priming [26–28]. There were no differences in baseline characteristics between studies, except for body surface area, which was higher in the crystalloid group [mean difference (MD): −0.03; 95% CI: −0.04, −0.01 m^2^; *P *<* *0.001]. Several investigators reported higher COPs at bypass initiation or after aortic cross-clamping in the RAP group than in the crystalloid group [27, 28] (*P *<* *0.001 and 14.6 ± 2.0 vs 12.5 ± 1.7 mmHg, *P *<* *0.05). However, COP was restored to baseline 6 h postoperatively without differences between groups [28].

### Haemodilution and fluid balance

#### Crystalloids versus colloids

**Figure 3:**
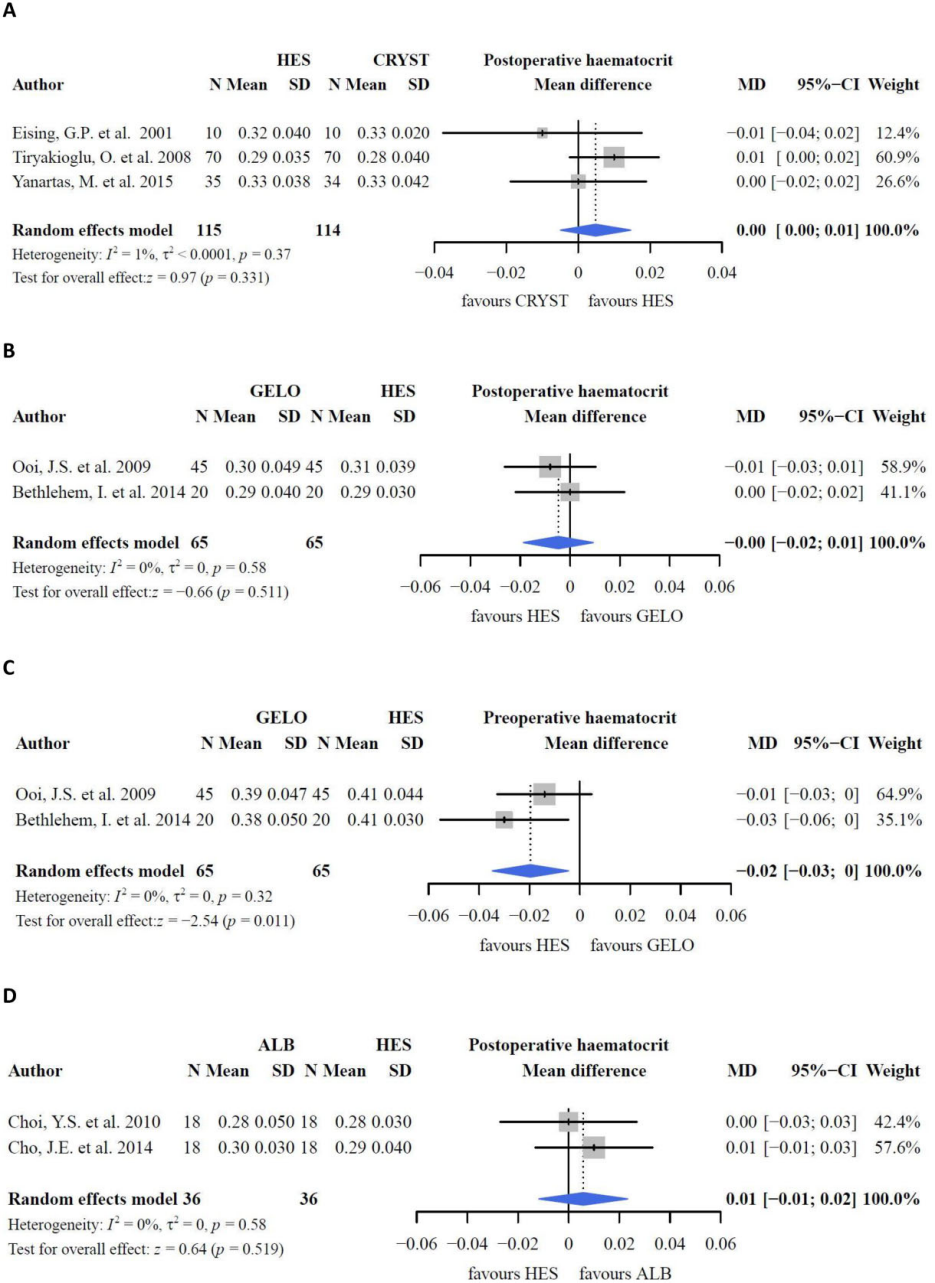
Haematocrit.

**Figure 4:**
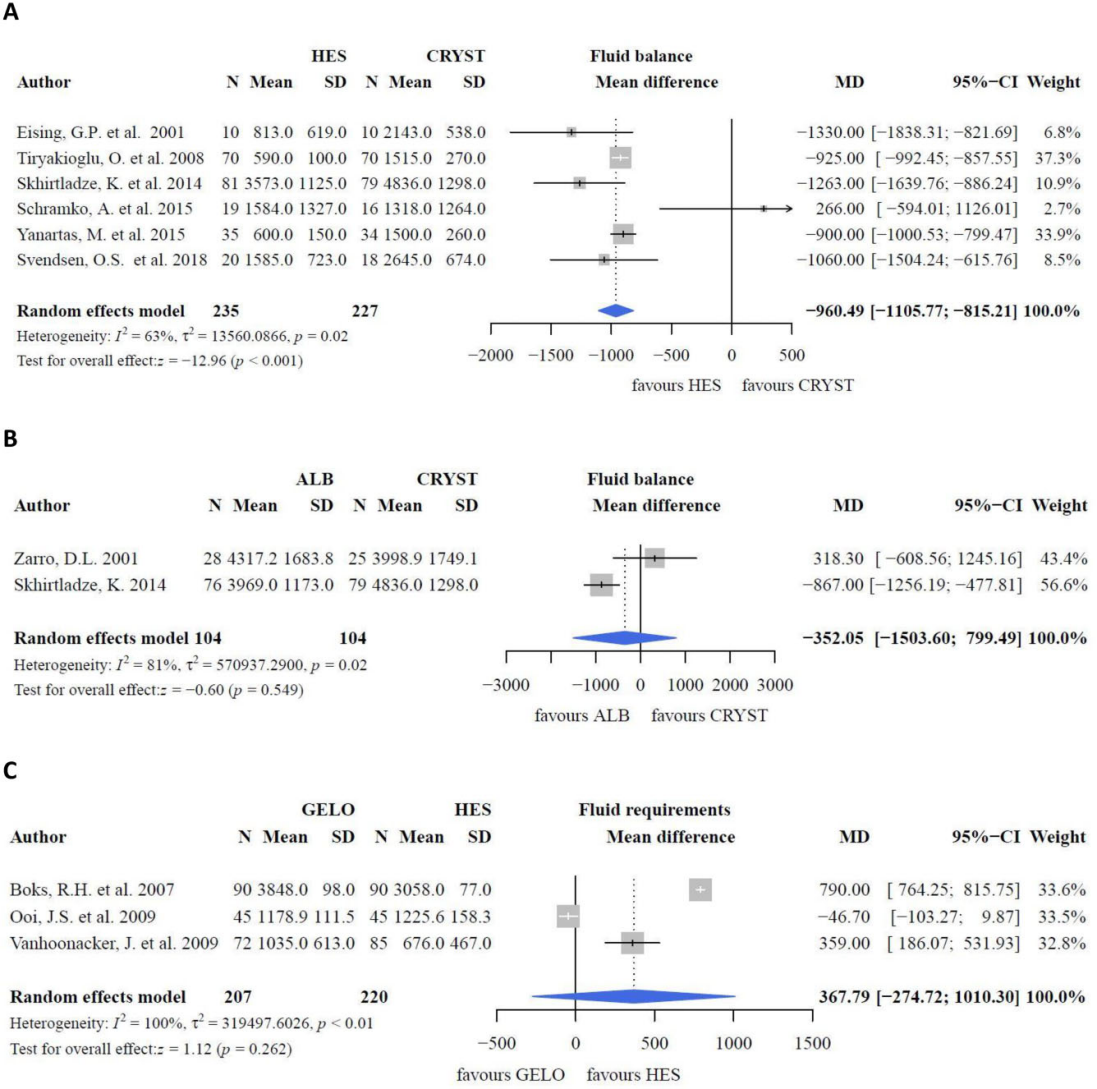
Fluid balance and fluid requirements.

In studies comparing crystalloids with colloids, no difference in postoperative haematocrit was found (Fig. 3A, P* *=* *0.331) [20, 29–31]. PRBC requirements increased with albumin and HES compared to LR in a study by Skhirtladze *et al.* [32] (*P *=* *0.0013). The study solution was used during anaesthesia induction (250–500 ml), CPB circuit (1500 ml) and intra- and postoperative periods. Other studies reported no differences in the transfusion rates of PRBCs comparing albumin [29] or gelofusine [19] with crystalloids. Fluid balance was lower with HES than with crystalloids (Fig. 4A; MD: −960.49; 95% CI: −1105.77, −815.21 ml; *P *<* *0.001) [20, 30–34]. Two studies reported an increased fluid balance with crystalloids compared to albumin [17, 32]. However, the pooled effect between albumin and crystalloids was not different (Fig. 4B, P* *=* *0.549) [29, 32]. One study could not be included in the meta-analysis owing to the different extent of data [17]. Fluid requirements increased with crystalloids compared with albumin [32] and HES [30–32]. Unfortunately, data could not be pooled because of an incomparable data format. The effects of colloid and crystalloid CPB priming on weight gain as a clinical outcome parameter remained inconsistent (HES versus crystalloids: −0.3 ± 1.5 vs 1.5 ± 1.2, *P *<* *0.05; albumin versus crystalloids: not significant) [20, 29].

#### Colloids versus colloids

**Figure 5:**
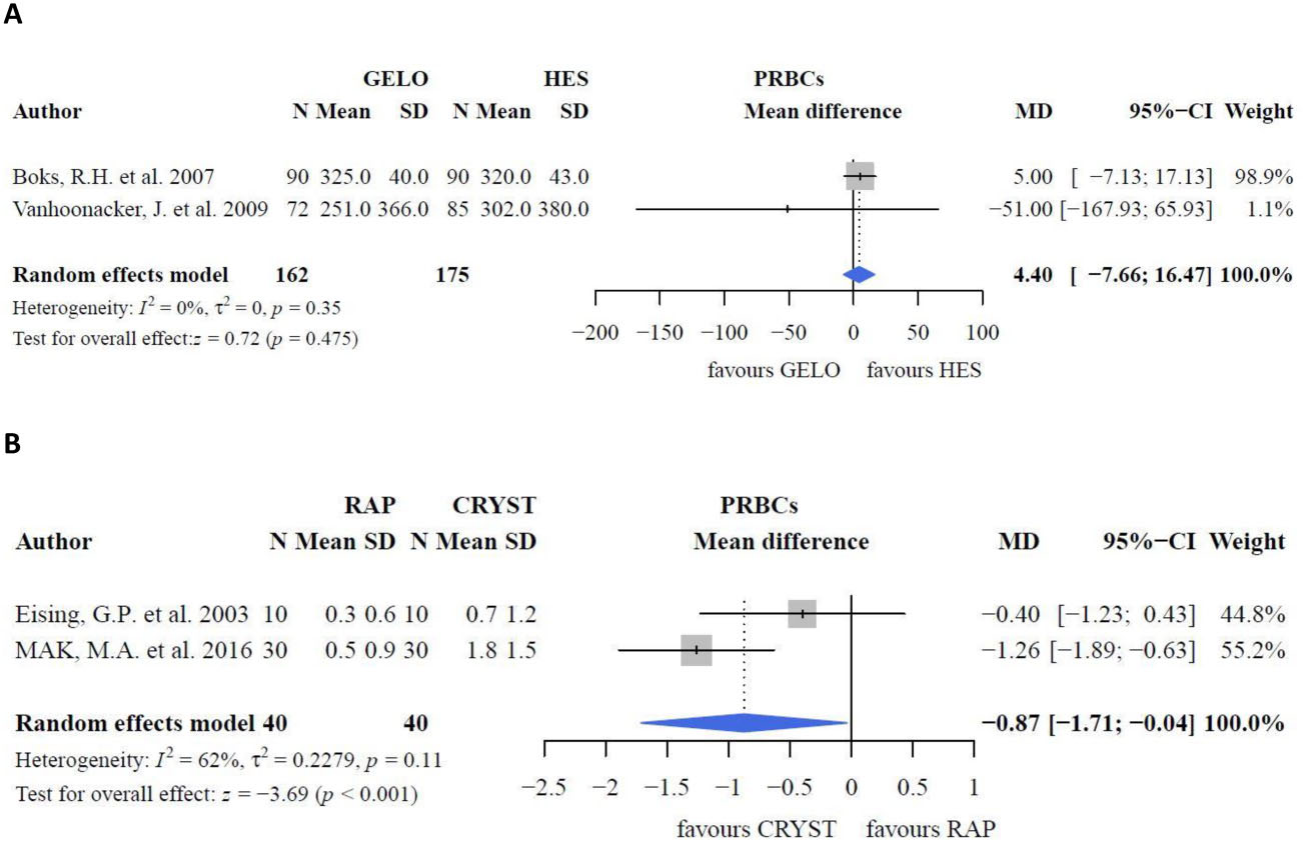
Packed red blood cell requirements.

In studies investigating HES and gelofusine as part of CPB priming, postoperative haematocrit levels were comparable (Fig. 3B, P* *=* *0.511), despite a higher preoperative haematocrit level with HES (Fig. 3C, P* *=* *0.011) [22, 35]. This finding is supported by studies in which gelofusine [25] and HES (Fig. 3D, P* *=* *0.519) [36, 37] were compared to albumin. Pooled differences in fluid- and PRBC requirements between HES and gelofusine were not significant (Figs. 4C and 5A) [21, 35, 38]. Similarly, intraoperative fluid balance and fluid requirements were comparable between albumin and HES, although these results could not be pooled in this meta-analysis [32, 36, 37].

#### Retrograde autologous priming

In a study where a CPB circuit was almost completely filled with RAP (620 ml + 30 ml LR), a smaller decrease in postoperative (24 h) haematocrit was reported compared with crystalloid CPB priming (650 ml) (*P *=* *0.001) [26]. Postoperative haematocrit remained similar when RAP was diluted in a larger CPB priming volume (450 ml + 650 ml LR) compared to crystalloids (1100 ml) [27]. Nevertheless, haematocrit after bypass initiation and unclamping was higher in the RAP + LR group (*P *<* *0.01 and *P *<* *0.05, respectively) [27]. Total PRBC requirements were higher in the crystalloid group (Fig. 5B; MD: −0.87; 95% CI: −1.71, −0.04 units; *P *<* *0.001). Lower PRBC requirement was reported when crystalloids (220 ml) and albumin (5% 100 ml) were combined with RAP (880 ml) than when crystalloids (1100 ml) and albumin (5% 100 ml) were used alone (*P *=* *0.03), although the units per patient transfused were not significantly different [39]. Intraoperative fluid balance [28] and fluid requirements [27] were higher in the crystalloid group than in the RAP group, although results could not be pooled because of a lack of studies. Postoperative weight gain (36 h) was lower in the RAP group than in the crystalloid group (0.1 ± 0.9 versus 1.5 ± 1.2 kg; *P *=* *0.05) [28].

### Haemostasis, blood loss and thrombocyte transfusion requirements

#### Crystalloids versus colloids

**Figure 6:**
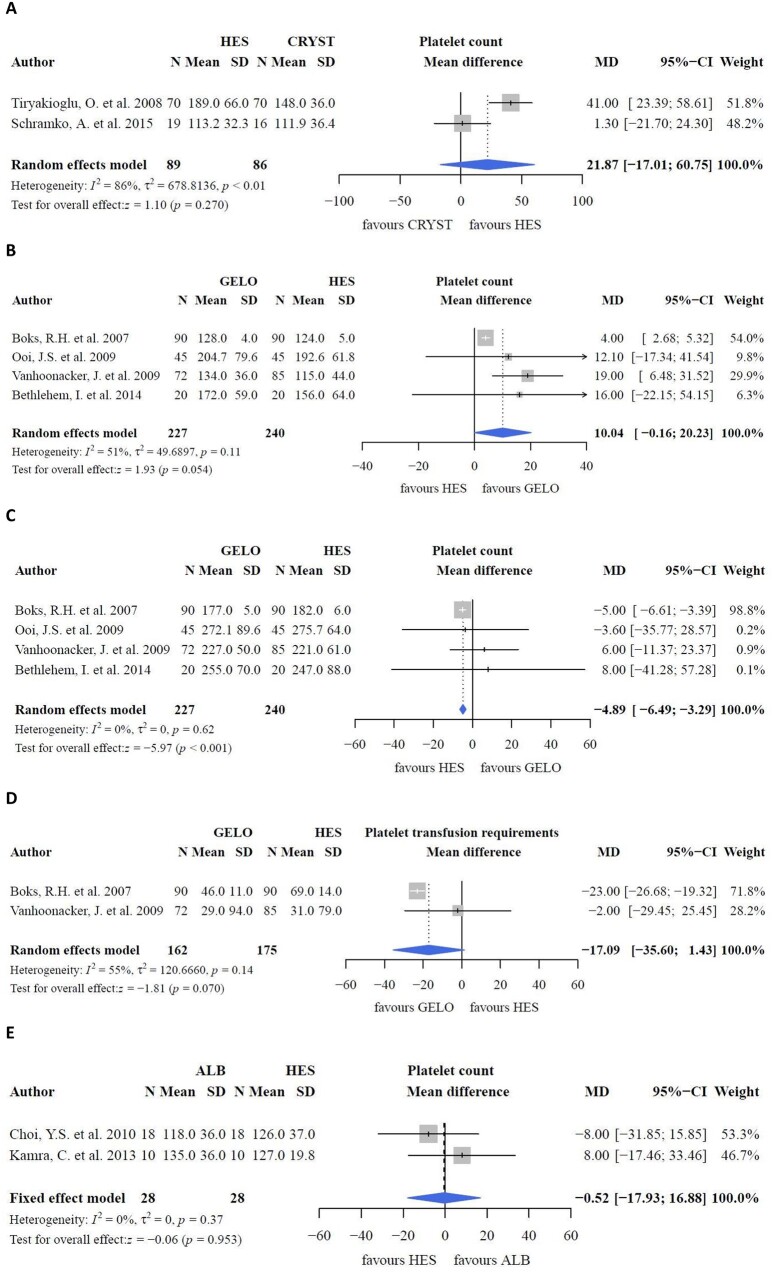
Platelet count and platelet transfusion requirements.

**Figure 7:**
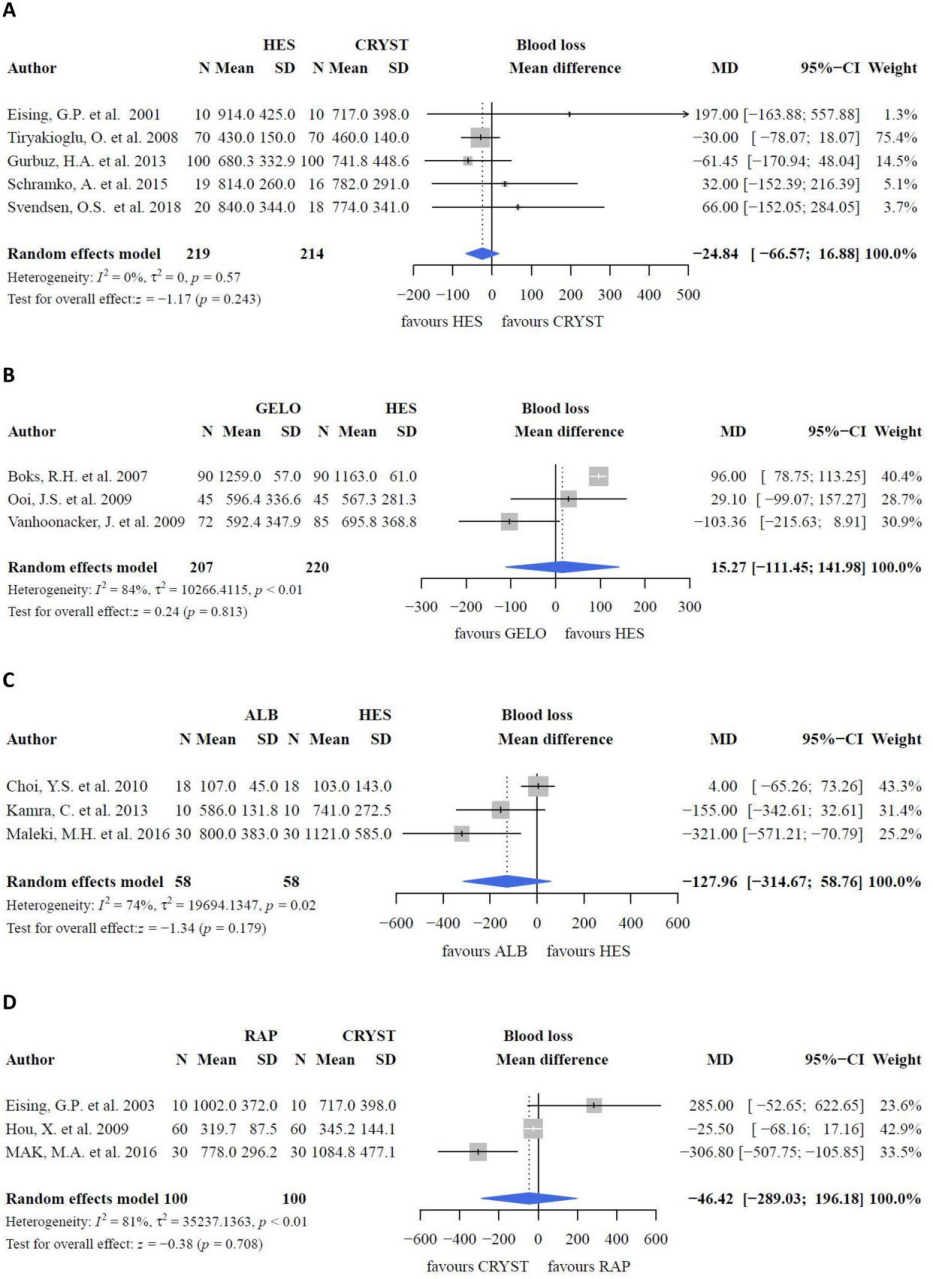
Blood loss. ALB: albumin; CRYST: crystalloids; GELO: gelofusine; HES: hydroxyethyl starch; RAP: retrograde autologous priming.

In a study comparing albumin, HES and LR as CPB priming fluids, both colloids had a negative impact on platelet count upon intensive care unit arrival (*P *<* *0.0001); ‘any blood product’ was higher in the colloid group (*P *=* *0.0003) than in the crystalloid group. However, blood loss, which was the primary outcome herein, did not differ between the groups (*P *=* *0.085). Moreover, blood loss was comparable in a study comparing gelofusine with crystalloids [19]. However, a negative effect of HES on platelets in the postoperative phase compared to crystalloids has been reported (*P *=* *0.001) [30], although the pooled effect was not different (Fig. 6A; *P *=* *0.270). Despite decreased platelet counts, platelet transfusion rates were conflicting. One study reported an increased platelet transfusion rate in priming groups with HES (*P *=* *0.024) [33]. Gurbuz *et al.* [40] reported an increased platelet transfusion rate in the crystalloid (Isolyte-M) group (*P *=* *0.035). However, no differences in platelet transfusion rates were reported between the groups [30, 31]. Moreover, the negative effects of albumin on platelet transfusion requirements compared to crystalloids were contradicting [29]. Herein, no differences in blood loss (Fig. 7A; *P *=* *0.243) were found between HES and crystalloids.

#### Colloids versus colloids

When colloids were compared as part of CPB priming, postoperative platelet counts did not differ between HES and gelofusine prime fluid (Fig. 6B; MD: 10.04; 95% CI: −0.16, 20.23 × 10^9^ l^−1^; *P *=* *0.054), despite a higher platelet count at baseline with HES (Fig. 6C; MD: −4.89; 95% CI: −6.49, −3.29 × 10^9^ l^−1^; *P *<* *0.001). Platelet transfusion requirements were also comparable between HES and gelofusine (Fig. 6D; MD: −17.09; 95% CI: −35.50, 1.43 ml; *P *=* *0.070). However, 1 study reported a greater decrease in platelet count (ΔPlt) with HES than that with albumin (mean ΔPlt: −142.36 [107.21] versus −48.28 [65.56] × 10^9^ l^−1^, *P *=* *0.007) [41]. Still, platelet transfusion requirements did not differ between the groups [36, 37, 41, 42]. Meanwhile, blood loss was comparable (Fig. 7B; *P *=* *0.813) between gelofusine and HES. Blood loss was higher with HES than with albumin in some studies (*P *=* *0.017 [41] and *P *<* *0.05 [43]), but did not differ in other studies [24, 37, 42]. Results of 2 of these studies [24, 42] could not be pooled because the data were reported as median ± range. The pooled effect showed no differences between albumin and HES with respect to blood loss (Fig. 7C; *P *=* *0.179) and postoperative platelet count (Fig. 6E; *P *=* *0.953). One study compared the incidence of bleeding between albumin and gelofusine but found no differences between them [24].

#### Retrograde autologous priming

CPB priming with RAP resulted in postoperative blood loss comparable with that of crystalloids only (Fig. 7D; MD: −46.42; 95% CI: −289.03, 196.18 ml; *P *=* *0.708) [26–28]. Platelet counts and platelet transfusion requirements were not reported.

## DISCUSSION

In this meta-analysis, COP did not differ between crystalloid and colloid or between 2 colloid CPB priming(s). In addition, fluid balance was lower with HES than with crystalloids. Fluid balance and fluid requirements were comparable between albumin, gelofusine and RAP in combination with crystalloids versus crystalloids alone. Haematocrit levels and PRBC transfusion requirements did not differ between colloid and crystalloid or colloid groups. However, the addition of RAP reduced PRBC transfusion requirements during bypass compared to crystalloid priming fluid alone, confirming the literature on RAP [44]. Finally, no differences in platelet count and blood loss were found between groups.

### Colloid oncotic pressure as a haemodilution parameter

COP may be considered a reliable indicator of haemodilution during cardiac surgery. The degree of haemodilution is determined by the amount and type of priming fluids for CPB and perioperative fluid therapy. All plasma proteins that serve to determine oncotic pressure in the intra- and extravascular compartments are represented by the COP, shown in mmHg. COP plays a key role in transcapillary fluid movement. Originally, transcapillary fluid movement was believed to be determined between 2 opposing forces: the hydrostatic pressure gradient (capillary pressure minus interstitial fluid pressure) versus the COP gradient (capillary COP minus interstitial fluid COP) [45]. However, the revised Starling equation states that net fluid movement across the capillary membrane is less affected by the interstitial fluid COP, and fluid is not absorbed by capillaries COP [46]. In contrast, the endothelial glycocalyx layer partly determines transcapillary fluid movement. It is the COP in the subglycocalyx that determines transcapillary flow [47]. The significance of the revised Starling principle is that a low plasma COP is associated with increased transcapillary fluid movement, resulting in tissue oedema [48]. However, the threshold at which oedema occurs remains controversial. Previous studies reported that low COP (<15 mmHg) during cardiac surgery was correlated with fluid overload [49], increased blood loss [49], increased postoperative weight gain [49, 50], prolonged mechanical ventilation duration [49, 51] and increased length of hospital stay [19, 49, 51]. However, a major limitation of previous studies is that the perioperative use of crystalloid and colloid fluids for resuscitation has not been consistently reported, except by Jansen, Te Velthuis [19]. Importantly, the choice for crystalloids compared with colloids or with RAP in CPB priming affects the perioperative change in COP. However, the pooled results of this meta-analysis showed no differences in COP between crystalloids and colloids. To compare the effect of COP between nothing but colloids is difficult, since colloid groups were usually a mixture of crystalloid and colloid fluid, rarely colloids alone. Also, it cannot be excluded that other factors may affect COP perioperatively. Besides crystalloids or colloids, factors that contribute to plasma COP include fibrinogen (0.35 mmHg) and free haemoglobin [52]. Intravascular haemolysis and thus increased free haemoglobin levels have been consistently reported during cardiac surgery with CPB [53]. However, the clinical implications of this mechanism and its role in COP require a more comprehensive evaluation before further conclusions can be drawn. The addition of RAP to CPB priming may reduce the need for red blood cell transfusion requirement, as shown herein. The increased transfusion requirement could be a result of earlier reached transfusion triggers owing to a more profound haemodilution with crystalloids than with crystalloids in combination with RAP(26, 27). This confirms a previous meta-analysis which reported higher haematocrit levels during bypass in combination with reduced transfusion requirements in RAP as part of CPB priming than in non-RAP(13). However, these results should be interpreted with some caution because the priming fluid types used in these studies were not mentioned. Moreover, the sample sizes of the included studies were small, and suspected bias across the included trials was extant, reflected by the low median Jadad scores.

### Net transcapillary fluid movement

This meta-analysis observed that fluid extravasation was lower with RAP or colloids, because intraoperative fluid balance [17, 19, 20, 27, 28, 30–34] and intraoperative fluid requirements [19, 27, 28, 30–32] were lower than those with crystalloids. This effect was more pronounced with HES (Fig. 4A) than with albumin (Fig. 4B) and gelofusine compared with crystalloids. This could be explained by the lower number of included studies (albumin *n* = 2, gelofusine *n* = 1, HES *n* = 6). The need for PRBC transfusions to compensate for haemodilution did not increase in crystalloid groups compared with albumin [29], gelofusine [19] or HES [30, 31, 33, 34, 40]. Similarly, in trials comparing 2 colloids PRBC requirements did not differ between groups (HES versus albumin [36, 37, 42], HES versus gelofusine [22, 23, 35, 38]), with the exception of Skhirtladze *et al.* [32]. Herein, the negative effect of albumin and HES on PRBC requirements could be explained by more profound haemodilution of the colloid groups than of the crystalloid groups, since the transfusion trigger was reached earlier [32]. Hence, a smaller proportion of the crystalloids may have remained in the intravascular space, explained by the lowered COP and intravascular volume, which was reflected by increased fluid balance and fluid requirements compared to the colloids. Positive effects of a lower intraoperative fluid balance on clinical outcomes (clinical performance score, median hospital stay, and weight gain) have been consistently reported [19, 20, 28]. Thus, it could be beneficial to prevent high intraoperative fluid balance with respect to patient outcomes.

### Haemostasis, blood loss and thrombocyte transfusion requirements

With regard to blood loss, no differences were observed between groups. The decrease in postoperative platelet count [21, 22, 35, 38] and thrombocyte transfusion rates (*P *=* *0.070) [21, 38] were comparable between HES and gelofusine. However, a trend was observed in lower postoperative platelet count with HES compared with gelofusine (*P* = 0.054), with higher preoperative platelet counts with HES as prime fluid (*P* < 0.001) [21, 22, 35, 38]. This result contradicts the suggested non-inferiority of HES over gelatine fluids in the meta-analysis by Ghijselings, Himpe [10]. There are several possible explanations for this trend. Firstly, the duration of CPB and aortic cross-clamping time were lower in the gelofusine group, although differences seem clinically irrelevant. However, heterogeneity was high (Fig. 6B: *I*^2^ = 51%; Fig. 6D: *I*^2^ = 55%). Secondly, according to the literature, uncoated CPB systems result in lower platelet counts [54]. Yet, 75% of the studies used either coated CPB systems or coated oxygenators [21, 22, 38], while 1 study did not report coating [35]. These differences in platelet counts were not observed between albumin and HES. Four studies were excluded from pooling because of incomparable data extent, of which 1 study reported lower platelet counts with HES than with albumin [41]. No differences in platelet transfusion were found between HES and albumin [36, 37, 41, 42]. The molecular weight of HES may determine its effect on haemostasis, as blood loss, the rate of reoperation for bleeding, and transfusion amounts increased in a meta-analysis comparing high molecular HES (450/0.7 and 200/0.5) with albumin [9]. Caution is required when interpreting these findings, as haemostatic data coming from conventional coagulation tests are not addressed in this meta-analysis.

### Future perspectives

Haemodilution during cardiac surgery with CPB is inevitable. The type of fluid(s) for an optimal CPB priming strategy, that improves patient outcomes in cardiac surgery settings remains to be determined. The question whether COP is a valuable parameter for measuring the effect and degree of haemodilution remains.

### Strength and limitations

There are several strengths of this study. It was conducted according to a prospectively designed and published analysis plan by a multidisciplinary group, with experience in cardiothoracic surgery, extracorporeal circulation, and cardiothoracic anaesthesiology. Studies were assessed by 2 independent reviewers who were blinded to each other’s results, and a third independent reviewer resolved any discrepancies. Furthermore, 7 priming strategies were systematically reviewed and analysed. Finally, 90% of the included studies were RCTs. Nevertheless, this meta-analysis has also some limitations. First, priming volume, cardioplegia volume and perioperative fluid volumes were not included, although this (partly) determines the degree of haemodilution during CPB and is a potential confounder. Second, not all studies were used for pooling owing to a lack of uniformity in data. Third, large statistical and clinical heterogeneity was relatively common in our study. This heterogeneity probably exists because of the variety of populations in the studies, a small number of studies included in some meta-analyses, different definitions used (e.g. transfusion triggers during CPB), and differences in protocols for clinical practise. There were no differences in baseline characteristics, except for CPB time, aortic cross-clamping time, age (HES versus gelofusine) and body surface area (RAP versus crystalloids). These differences seem clinically irrelevant (Supplementary Material, Table S2). A random-effects model was used to incorporate heterogeneity among studies. Heterogeneity presumably exists with a low number of included studies, whether or not it was statistically detected (*I*^2^). Therefore, in meta-analyses with low statistical heterogeneity, random-effects model was used to account for the low number of included studies. Although, at least 2 studies are sufficient to perform a meta-analysis [55], the result of data pooling should be interpret with some caution. Another relevant limitation is that the effect of COP, haemodilution and haemostasis on patient outcome is not addressed in this meta-analysis. Moreover, during initial screening, not all studies were available in full text. Finally, it is possible that eligible articles were not identified using our search strategy.

## CONCLUSION

In conclusion, no difference in COP was found between crystalloid and colloid priming solutions. In addition, different colloids were found to be non-inferior with respect to the decrease in COP during bypass. These results suggest that fluid extravasation is less determined by the type of CPB priming used. According to the currently available literature included in this meta-analysis, there is no optimal strategy for prime fluids to maintain COP with respect to crystalloids or colloids or with RAP for patients undergoing elective cardiac surgery with CPB.

## SUPPLEMENTARY MATERIAL

Supplementary material is available at *ICVTS* online.

## Supplementary Material

ivac127_Supplementary_Data

## Data Availability

Template forms, data and analytic codes are available upon request.

## ABBREVIATIONS

CI: Confidence interval
COP: Colloid oncotic pressure
CPB: Cardiopulmonary bypass
HES: Hydroxyethyl starch
LR: Lactated Ringer’s
MD: Mean difference
PRBCs: Packed red blood cells
RAP: Retrograde autologous priming
RCT: Randomized controlled trial
