# Supplementary material for: Effects of crystalloid and colloid priming strategies for cardiopulmonary bypass on colloid oncotic pressure and haemostasis: a meta-analysis
Source: Interact Cardiovasc Thorac Surg. 2022 May 5;35(3):ivac127. doi: 10.1093/icvts/ivac127 (PMC9419694; doi:10.1093/icvts/ivac127)
Supplement: ivac127_Supplementary_Data [file ivac127_supplementary_data.zip › ivac127_Supplementary_Data/ICVTS Supplementary online content Beukers AM 04.2022.docx]

| **Supplementary online content**  Appendix 1. Risk of bias  Supplementary Figure 1. Summary of the risk of bias among the randomised controlled trials using the Cochrane Risk of Bias tool  Supplementary Figure 2. Summary of the risk of bias among the observational trials using the ROBINS-I tool  Supplementary Table 1. Search strategy  Supplementary Table 2. Differences in the baseline characteristics of the included studies  Supplementary Table 3. Summary of the included studies |
| --- |

**Appendix 1. Risk of bias**

The randomised controlled studies were analysed using the Cochrane Risk of Bias tool (Fig. 2) and the non-randomised controlled studies using the ROBINS-I tool (Fig. 3). The judgement of bias was focused on selection bias, performance bias, detection bias, attrition bias, reporting bias, confounding, and other possible sources of bias. The risk of bias in the different domains was judged and classified as high, low, or unclear. The risk of bias for an individual study was categorised as low when the risk of bias was low in all domains; unclear when the risk of bias was unclear in at least one domain, with no high-risk domains; and high when the risk of bias was high in at least one domain.

*Cochrane Risk of Bias tool*

*Selection bias:* The randomisation method was described in all studies. Six studies described a non-random component in the generation process and were considered inadequate(1-6). Allocation concealment prior to assignment was adequate in seven studies(7-13). Thirteen studies did not mention allocation concealment(2, 4, 14-24).

*Performance and detection bias:* Proper blinding of participants and personnel was achieved in 46% of the included randomised controlled trials(5, 7, 8, 10-12, 15-18, 23, 25). When only the investigator was blinded, not the perfusionist, the study was still assessed as having a low risk of bias. Blinding of outcome assessment was ensured in only 3 studies(7, 9, 23) and not mentioned in 18 studies(4, 5, 8, 10-15, 17-22, 24-26).

*Attrition bias:* Four out of twenty-six studies did not mention the reasons for dropouts or missing outcomes; thus, the attrition bias was considered high(1-3, 25). The vast majority of the included studies substantiated their missing outcomes and were assessed as adequate(4, 5, 7-24, 26, 27).

*Reporting bias:* In 92% of the randomised controlled trials, all of the pre-specified outcomes of interest were reported. Therefore, selective reporting was considered low(1-5, 7-14, 16-20, 22-24, 26, 27).

*Other bias:* Seven studies presented non-response bias owing to the small sample size(1, 4, 18, 22-24, 27). In three studies, no sample size calculation was performed; thus, they were considered to have a high risk of bias(1, 2, 14). In one study, the risk of other bias could not be determined owing to premature discontinuation of the study(25).

*ROBINS-I tool*

Bias in the classification of interventions, bias due to deviations from intended interventions, bias in the measurement of outcomes, and bias in the selection of the reported results were considered low in all observational trials(28-30). Bias due to confounding was judged as a critical risk in the study by Tiryakioglu, Yildiz (29). In the studies by Tiryakioglu, Yildiz (29) and Zarro, Palanzo (30) there was a critical risk of bias in the selection of participants, for instance, by not mentioning their inclusion and exclusion criteria. Bias due to missing data was considered a moderate risk in only one study(30).


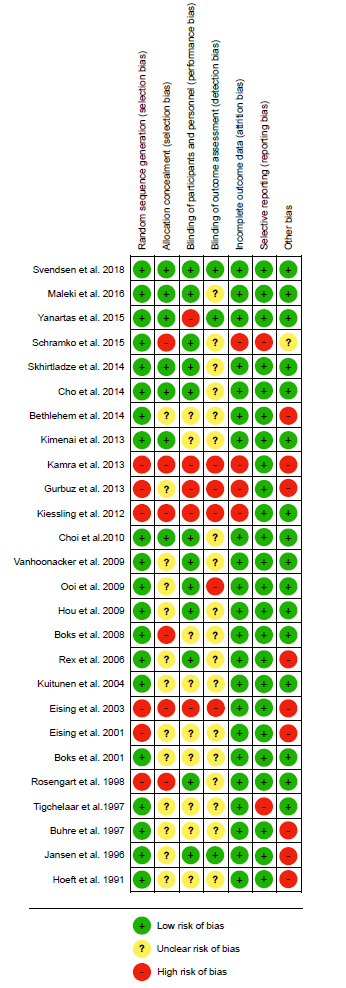


Supplementary Figure 1. Summary of the risk of bias among the randomised controlled trials using the Cochrane Risk of Bias tool


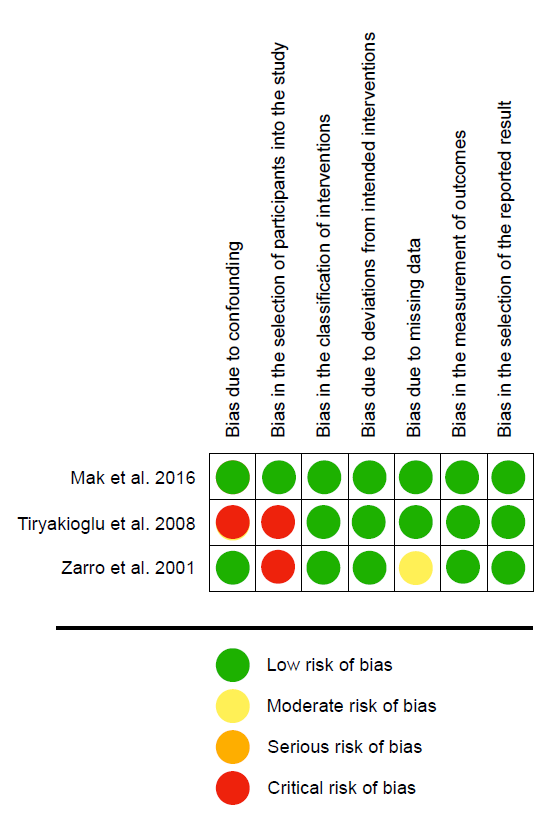


Supplementary Figure 2. Summary of the risk of bias among the observational trials using the ROBINS-I tool

Supplementary Table 1. Search strategy

| **Search** | **PubMed and Embase** |
| --- | --- |
| #1 | “Cardiac Surgical procedures” or cardiac surg* or coronary artery bypass* or cardiac operat* or heart surg* or heart operat* or cardiosurg* |
| #2 | “Extracorporeal Circulation” or “Heart-Lung Machine” or “Cardiopulmonary Bypass” or extracorporeal circulation or heart-lung machine* or cardiopulmonary bypass* or heart-lung bypass* |
| #3 | “Colloids” or “Albumins” or “Serum Albumin” or “succinylated gelatin” or “isotonic solutions” or “Crystalloid Solutions” or colloid* or crystalloid* or albumin* or gelofusine* or succinylated gelatin* or ringer’s lactat* or ringer’s solution* or ringer lactat* or osmolit* |
| #4 | #1 and #2 and #3 |
| #5 | NOT “Adolescent” or “Child” or “Infant” or adolescen* or child* or schoolchild* or infant* or girl* or boy* or teen* or teens or teenager* or youth* or pediatr* or paediatr* or puber* NOT “Adult” or adult* or man or men or woman or women |

Supplementary Table 2. Differences in the baseline characteristics of the included studies

| Covariate per subgroup | Number of trials reporting the variable (%) | I^2^ | MD (95% CI) | *P*-value |
| --- | --- | --- | --- | --- |
| Albumin versus crystalloids | | | | |
| Age (year) | 3 (60%) | 0% | 0.11 (-3.16; 3.39) | 0.945 |
| BMI (kg m^-2^) | 0 (0%) | - | - | - |
| BSA (m^2^) | 4 (80%) | 0% | 0.05 (-0.01; 0.11) | 0.104 |
| CPB time (min) | 4 (80%) | 0% | 6.23 (-5.26; 17.72) | 0.288 |
| AoX time (min) | 4 (80%) | 0% | 4.62 (-4.02; 13.27) | 0.295 |
| EuroSCORE | 0 (0%) | - | - | - |
| HES versus albumin | | | | |
| Age (year) | 4 (57%) | 29% | 2.18 (-1.37; 5.73) | 0.229 |
| BMI (kg m^-2^) | 3 (43%) | 0% | -0.27 (-1.22; 0.68) | 0.581 |
| BSA (m^2^) | 0 (0%) | - | - | - |
| CPB time (min) | 5 (71%) | 6% | 1.32 (-4.34; 6.97) | 0.648 |
| AoX time (min) | 5 (71%) | 0% | 0.67 (-3.73; 5.08) | 0.764 |
| EuroSCORE | 0 (0%) | - | - | - |
| HES versus gelofusine | | | | |
| Age (year) | 5 (83%) | 86% | 0.98 (0.69; 1.28) | <0.001^*^ |
| BMI (kg m^-2^) | 1 (17%) | - | - | - |
| BSA (m^2^) | 3 (50%) | 0% | 0.00 (-0.01; 0.01) | 0.978 |
| CPB time (min) | 5 (83%) | 84% | -4.84 (-6.27; -3.41) | <0.001^*^ |
| AoX time (min) | 5 (83%) | 89% | -3.70 (-4.79; -2.61) | <0.001^*^ |
| EuroSCORE | 2 (33%) | 0% | 0.30 (-0.25; 0.85) | 0.281 |
| HES versus crystalloids | | | | |
| Age (year) | 6 (86%) | 29% | -0.54 (-2.03; 0.96) | 0.483 |
| BMI (kg m^-2^) | 2 (29%) | 35% | 0.59 (-0.27; 1.45) | 0.176 |
| BSA (m^2^) | 2 (29%) | 0% | -0.00 (-0.11; 0.11) | 0.972 |
| CPB time (min) | 6 (86%) | 69% | 7.07 (-1.28; 15.42) | 0.097 |
| AoX time (min) | 6 (86%) | 72% | 3.3 (-3.27; 9.96) | 0.321 |
| EuroSCORE | 2 (29%) | 0% | 0.22 (-0.65; 1.08) | 0.625 |
| Retrograde autologous priming versus crystalloids | | | | |
| Age (year) | 3 (100%) | 48% | 1.41 (-0.43; 3.24) | 0.133 |
| BMI (kg m^-2^) | 0 (0%) | - | - | - |
| BSA (m^2^) | 2 (67%) | 0% | -0.03 (-0.04; -0.01) | <0.001^*^ |
| CPB time (min) | 3 (100%) | 42% | 0.60 (-5.40; 6.60) | 0.845 |
| AoX time (min) | 3 (100%) | 32% | -2.20 (-6.79; 2.39) | 0.348 |
| EuroSCORE | 0 (0%) | - | - | - |

Number of trials reporting the baseline characteristics. Significant differences were measured using a weighted fixed effects model. When the I^2^ was >50%, a random effects model was used. Some baseline characteristics were reported in medians with interquartile ranges and were therefore incomparable. The baseline characteristics were not significantly different among the studies comparing between gelofusine and crystalloids(23) and between gelofusine and albumin(21, 26). Owing to a different extent of data, the baseline characteristics were not comparable among these studies. Abbreviations: Aortic Cross Clamping (AoX), Body Surface Area (BSA), Body Mass Index (BMI), European system for cardiac operative risk evaluation (EuroSCORE).

**References**

1. Kamra C, Beney A. Human albumin in extracorporeal prime: effect on platelet function and bleeding. Perfusion. 2013 Nov;28(6):536-40.

2. Gurbuz HA, Durukan AB, Salman N, Tavlasoglu M, Durukan E, Ucar HI, et al. Hydroxyethyl starch 6%, 130/0.4 vs. a balanced crystalloid solution in cardiopulmonary bypass priming: a randomized, prospective study. J Cardiothorac Surg. 2013 Apr 8;8:71.

3. Kiessling AH, Wedde S, Keller H, Reyher C, Stock U, Beiras-Fernandez A, et al. Pre-filling of the extracorporeal circuit with autologous blood is safe, but not effective in optimizing biocompatibility in high-risk patients. Perfusion. 2012 Sep;27(5):371-7.

4. Eising GP, Niemeyer M, Gunther T, Tassani P, Pfauder M, Schad H, et al. Does a hyperoncotic cardiopulmonary bypass prime affect extravascular lung water and cardiopulmonary function in patients undergoing coronary artery bypass surgery? Eur J Cardiothorac Surg. 2001 Aug;20(2):282-9.

5. Rosengart TK, DeBois W, O'Hara M, Helm R, Gomez M, Lang SJ, et al. Retrograde autologous priming for cardiopulmonary bypass: a safe and effective means of decreasing hemodilution and transfusion requirements. J Thorac Cardiovasc Surg. 1998 Feb;115(2):426-38; discussion 38-9.

6. Eising GPP, M.; Niemeyer, M.; Tassani, P.; Schad, H.; Bauernschmitt, R.; Lange, R. Retrograde autologous priming: Is it useful in elective on-pump coronary artery bypass surgery? Ann Thorac Surg. 2003;75:23-7.

7. Svendsen OS, Farstad M, Mongstad A, Haaverstad R, Husby P, Kvalheim VL. Is the use of hydroxyethyl starch as priming solution during cardiac surgery advisable? A randomized, single-center trial. Perfusion. 2018 Sep;33(6):483-9.

8. Hosseinzadeh Maleki M, Derakhshan P, Rahmanian Sharifabad A, Amouzeshi A. Comparing the Effects of 5% Albumin and 6% Hydroxyethyl Starch 130/0.4 (Voluven) on Renal Function as Priming Solutions for Cardiopulmonary Bypass: A Randomized Double Blind Clinical Trial. Anesth Pain Med. 2016 Feb;6(1):e30326.

9. Yanartas M, Baysal A, Aydin C, Ay Y, Kara I, Aydin E, et al. The effects of tranexamic acid and 6% hydroxyethyl starch (HES) solution (130/0.4) on postoperative bleeding in coronary artery bypass graft (CABG) surgery. Int J Clin Exp Med. 2015;8(4):5959-71.

10. Skhirtladze K, Base EM, Lassnigg A, Kaider A, Linke S, Dworschak M, et al. Comparison of the effects of albumin 5%, hydroxyethyl starch 130/0.4 6%, and Ringer's lactate on blood loss and coagulation after cardiac surgery. Br J Anaesth. 2014 Feb;112(2):255-64.

11. Cho JE, Shim JK, Song JW, Lee HW, Kim DH, Kwak YL. Effect of 6% hydroxyethyl starch 130/0.4 as a priming solution on coagulation and inflammation following complex heart surgery. Yonsei Med J. 2014 May;55(3):625-34.

12. Choi YS, Shim JK, Hong SW, Kim JC, Kwak YL. Comparing the effects of 5% albumin and 6% hydroxyethyl starch 130/0.4 on coagulation and inflammatory response when used as priming solutions for cardiopulmonary bypass. Minerva Anestesiol. 2010 Aug;76(8):584-91.

13. Kimenai DM, Bastianen G, Daane CR, Megens- Bastiaanse CM, Van Der Meer NJM, Scohy TV, et al. Effect of colloids Gelatin and HES 130/0.4 on blood coagulation in cardiac surgery patients: A randomised controlled trial. Applied Cardiopulmonary Pathophysiology. 2013;17(2):140-1.

14. Bethlehem I, Wierda K, Visser C, Jekel L, Koopmans M, Kuiper MA. Influence of Two Colloidal Extracorporeal Primes on Coagulation of Cardiac Surgical Patients: A Prospectively Randomized Open-Label Pilot Trial. J Extra Corpor Technol. 2014 Dec;46(4):293-9.

15. Vanhoonacker J, Ongenae M, Vanoverschelde H, Donadoni R. Hydroxyethyl starch 130/0.4 versus modified fluid gelatin for cardiopulmonary bypass priming: the effects on postoperative bleeding and volume expansion needs after elective CABG. Acta Anaesthesiol Belg. 2009;60(2):91-7.

16. Ooi JS, Ramzisham AR, Zamrin MD. Is 6% hydroxyethyl starch 130/0.4 safe in coronary artery bypass graft surgery? Asian Cardiovasc Thorac Ann. 2009 Aug;17(4):368-72.

17. Hou X, Yang F, Liu R, Yang J, Zhao Y, Wan C, et al. Retrograde autologous priming of the cardiopulmonary bypass circuit reduces blood transfusion in small adults: a prospective, randomized trial. Eur J Anaesthesiol. 2009 Dec;26(12):1061-6.

18. Rex S, Scholz M, Weyland A, Busch T, Schorn B, Buhre W. Intra- and extravascular volume status in patients undergoing mitral valve replacement: crystalloid vs. colloid priming of cardiopulmonary bypass. Eur J Anaesthesiol. 2006 Jan;23(1):1-9.

19. Kuitunen AH, Hynynen MJ, Vahtera E, Salmenpera MT. Hydroxyethyl starch as a priming solution for cardiopulmonary bypass impairs hemostasis after cardiac surgery. Anesth Analg. 2004 2004;98(2):291-7, table of contents.

20. Boks RH, van Herwerden LA, Takkenberg JJ, van Oeveren W, Gu YJ, Wijers MJ, et al. Is the use of albumin in colloid prime solution of cardiopulmonary bypass circuit justified? Ann Thorac Surg. 2001 Sep;72(3):850-3.

21. Tigchelaar I, Gallandat Huet RC, Korsten J, Boonstra PW, van Oeveren W. Hemostatic effects of three colloid plasma substitutes for priming solution in cardiopulmonary bypass. Eur J Cardiothorac Surg. 1997 Apr;11(4):626-32.

22. Buhre W, Hoeft A, Schorn B, Weyland A, Scholz M, Sonntag H. Acute affect of mitral calve replacement on extravascular lung water in patients receiving colloid or crystalloid priming of cardiopulmonary bypass. Br J Anaesth. 1997 Sep;79(3):311-6.

23. Jansen PGM, Te Velthuis H, Wildevuur WR, Huybregts MAJM, Bulder ER, Van Der Spoel HI, et al. Cardiopulmonary bypass with modified fluid gelatin and heparin-coated circuits. British Journal of Anaesthesia. 1996;76(1):13-22.

24. Hoeft A, Korb H, Mehlhorn U, Stephan H, Sonntag H. Priming of cardiopulmonary bypass with human albumin or Ringer lactate: effect on colloid osmotic pressure and extravascular lung water. Br J Anaesth. 1991 Jan;66(1):73-80.

25. Schramko A, Suojaranta-Ylinen R, Niemi T, Pesonen E, Kuitunen A, Raivio P, et al. The use of balanced HES 130/0.42 during complex cardiac surgery; effect on blood coagulation and fluid balance: a randomized controlled trial. Perfusion. 2015 2015;30(3):224-32.

26. Boks RH, Wijers MJ, Hofland J, Takkenberg JJ, Bogers AJ. Low molecular starch versus gelatin plasma expander during CPB: does it make a difference? Perfusion. 2007 Sep;22(5):333-7.

27. Eising GP, Pfauder M, Niemeyer M, Tassani P, Schad H, Bauernschmitt R, et al. Retrograde autologous priming: is it useful in elective on-pump coronary artery bypass surgery? Ann Thorac Surg. 2003 Jan;75(1):23-7.

28. Mak MA, Smolka A, Kowalski J, Kuc A, Klausa F, Kremens K, et al. Can cardiopulmonary bypass system with blood priming become a new standard in coronary surgery? Kardiol Pol. 2016;74(8):726-32.

29. Tiryakioglu O, Yildiz G, Vural H, Goncu T, Ozyazicioglu A, Yavuz S. Hydroxyethyl starch versus Ringer solution in cardiopulmonary bypass prime solutions (a randomized controlled trial). J Cardiothorac Surg. 2008 Jul 12;3:45.

30. Zarro DL, Palanzo DA, Phillips TG. Albumin in the pump prime: its effect on postoperative weight gain. Perfusion. 2001 Mar;16(2):129-35.
