# Supplementary material for: Effects of crystalloid and colloid priming strategies for cardiopulmonary bypass on colloid oncotic pressure and haemostasis: a meta-analysis
Source: Interact Cardiovasc Thorac Surg. 2022 May 5;35(3):ivac127. doi: 10.1093/icvts/ivac127 (PMC9419694; doi:10.1093/icvts/ivac127)
Supplement: ivac127_Supplementary_Data [file ivac127_supplementary_data.zip › ivac127_Supplementary_Data/Supplementary Table 3. Summary of included studies.docx]

| Reference  Supplementary Table 3. Summary of included studies | N | Study design | Type cardiac surgery | Blood cardioplegia | Cell saver | Colloid group – type + dose (mL) | Crystalloid group – type + dose (mL) | Additional prime – type + dose (mL) | Total prime (mL) | RAP (ml) | Peri-operative fluid protocol | Received colloids in both groups | Relevant outcomes reported |
| --- | --- | --- | --- | --- | --- | --- | --- | --- | --- | --- | --- | --- | --- |
| Bethlehem et al. 2014 | 40 | RCT | CABG, Valve | Yes | Yes | 1: HES 130/0.4 6% 1300  2: modified gelatin 1300, KCl 7.45% 20, CaCl 2.25umol.L‾¹ 10 | None | Mannitol 20% 200, NaHCO³‐ 8.4% 50, tranexamic acid 2g | NR | No | NR | NR | COP, haematocrit, fluid balance (median + IQR), blood loss (median + IQR), PRBCs (median + IQR), platelets, platelet transfusion requirements (no data) |
| Boks et al. 2001 | 60 | RCT | CABG, Valve | No | No | 1: Alb 20g + gelofusine 1400  2: Alb 2g + gelofusine 1400  3: gelofusine 1400 | None | Mannitol 20% 200, heparin 7500 IU | NR | No | NR | NR | COP, haematocrit (no data), platelets (no data) |
| Boks et al. 2008 | 180 | RCT | CABG (+) Valve | No | No | 1: HES 130/0.4 1000, + LR 300-500  2: Gelatin plasma expansion 1300-1500 | None | Mannitol 20% 200, heparin 7500 IU | NR | No | NR | NR | COP, haematocrit (no SD), fluid balance, fluid requirements, blood loss, PRBCs, platelets, platelet transfusion requirements |
| Buhre et al. 1997 | 26 | RCT | Valve | No | No | Alb 20% 400 + LR 1000 | LR 1400 | Glucose 5% 500 + Bic 100 | 2000 | No | NR | NR | Weight |
| Cho et al. 2014 | 54 | RCT | CABG (+) valve, Aortic | No | Yes | 1: HES 130/0.4 6% 500 + plasmalyte 1000 (max 20 ml.kg‾¹.day‾¹ HES)  2: Alb 5% 500 + plasmalyte 1000 | None | Mannitol 20% 5mL.kg‾¹, NaHCO³‐ 40 mEq, heparin 10 mg.L‾¹ | NR | No | Yes | No | Haematocrit, fluid requirements (no data), blood loss (median + IQR), PRBCs (units/patient), platelets (no SD), platelet transfusion requirements (no SD) |
| Choi et al. 2010 | 36 | RCT | Valve | Yes, cold | Yes | 1: HES 130/0.4 6% 500 + additional LR  2: Alb 5% 500 + additional LR | None | Mannitol 20% 5mL.kg‾¹, NaHCO³‐ 40 mEq, heparin 10 mg.L‾¹ | 1600 | No | Yes | No | Haematocrit, fluid requirements, blood loss, PRBCs (no SD), platelets, platelet transfusion requirements |
| Eising et al. 2001 | 20 | RCT | CABG | No | No | HES 10% 1100 | LR 1100 | Mannitol 20% 3 ml.kg‾¹, NaHCO³‐ 4.2% 5 ml/kg‾¹, Inzolen 20, heparin 5000 IU | NR | No | NR | NR | COP, haematocrit, fluid balance, weight gain, blood loss. |
| Eising et al. 2003 | 20 | RCT | CABG | No | No | None | LR 1100 | Mannitol 3ml.kg‾¹, potassium 5 mval, heparin 5000 IU | NR | Yes ±200 | NR | NR | COP, fluid balance, weight gain, blood loss |
| Gurbuz et al. 2013 | 200 | RCT | CABG | Yes, cold | No | HES 130/0.4 6% + NaCl 0.9% | Isolyte M | Heparin 5000 IU | 1500 | No | NR | NR | Blood loss, PRBCs, platelet transfusion requirements |
| Hoeft et al. 1991 | 20 | RCT | CABG | No | No | Alb 20% 400 + LR 1000 | LR 1400 | Glucose 5% 500 + Bic 100 | 2000 | No | NR | NR | COP |
| Hou et al. 2009 | 120 | RCT | CABG (+) Valve, aortic | Yes, cold | No | None | Acetated Ringer’s 1000 | NaHCO³‐ 30 mEq, heparin 5000 IU | ±1100 | Yes 150 -650 | NR | NR | COP, haematocrit, fluid requirements, blood loss, PRBCs, platelet transfusion requirements |
| Jansen et al. 1996 | 20 | RCT | CABG | No | No | Modified fluid gelatin 1000 + LR 500 | LR 1500 | Mannitol 20% 100, NaHCO³‐ 8.4% 50, heparin 500 IU | 1650 | No | Yes | Yes | COP, fluid balance, fluid requirements, blood loss, PRBCs (Units + IQR), platelets |
| Kamra et al. 2013 | 20 | RCT | CABG | Yes, cold | No | 1: HES 130/0.4 6% 500 + Plasma-Lyte A 1100  2: Alb 5% 50 + HES 130/0.4 6% 500 + Plasma-Lyte A 1050 | None | Mannitol 20% 150 + NaHCO³‐ 7.5% 50, heparin 10000 IU | 1800 | No | NR | NR | Blood loss, platelets |
| Kiessling et al. 2012 | 72 | RCT | CABG (+) Valve, | NR | Yes | 1: HES 6% 500 + electrolytic solution 200 + crystalloid 200-400  2: HES 6% 500 + electrolytic solution 200 + RAP | None | Mannitol 20% 200, tranexamic acid 20 | 1250 | Yes 200-400 | NR | NR | PRBCs, platelet transfusion requirements |
| Kimenai et al. 2013 | 60 | RCT | CABG | Yes, warm | No | 1: HES 130/0.4 6% 550-650 + LR 550-650  2: gelatin 550-650, LR 550-650 | None | Mannitol 20% 100, heparin 7500 IU | 1200-1400 | No | Yes | No | COP, blood loss (median + IQR), PRBCs (median + IQR), platelets (IQR), platelet transfusion requirements (no data) |
| Kuitunen et al. 2014 | 45 | RCT | CABG | No | No | 1: HES 120/0.7 20 ml.kg‾¹ + LR  2: Alb 5% 20 ml.kg‾¹ + LR | None | Heparin 5000 IU | 2000 | No | Yes | Yes | Fluid requirements (median + IQR), blood loss (median + IQR), platelets (no SD), platelet transfusion requirements (median + IQR) |
| MAK et al. 2016 | 60 | Pros. cohort | CABG | No | No | None | Crystalloid 650 | NR | 650 | Yes 650 | NR | NR | Haematocrit, blood loss, PRBCs |
| Maleki et al. 2016 | 60 | RCT | CABG | No | No | 1: HES 130/0.4 6% 500 + NaCl 0.9% 1000  2: Alb 5% 500 + NaCl 0.9% 1000 | None | Mannitol 20% 5mL.kg‾¹, NaHCO³‐ 45 mEq, heparin 10 mg.L‾¹ | 1500 | No | NR | NR | Blood loss, PRBCs, platelets, platelet transfusion requirements |
| Ooi et al. 2009 | 90 | RCT | CABG | Yes, cold | No | 1: HES 130/0.4 6%  2: succinylated gelatin 4% | None | Mannitol 0.5g.kg‾¹ | 1600 | No | Yes | No | Haematocrit, fluid requirements, blood loss, PRBCs, platelets, platelet transfusion requirements |
| Rex et al. 2006 | 22 | RCT | Valve | No | Yes | Alb 20% 400 + LR 1600 | LR 2000 | NR | 2000 | No | Yes | No | COP, fluid balance |
| Rosengart et al. 1998 | 60 | RCT | CABG | Yes, cold |  | 1: Serum albumin 100 + Plasma-Lyte 7.4 1100  2: Serum albumin 100 + Plasma-Lyte 7.4 1100 + RAP | None | Mannitol 20% 200, heparin 10000 IU | 1400 | Yes up to 1000 | NR |  | Fluid requirements, weight gain, PRBCs, platelet transfusion requirements |
| Schramko et al. 2015 | 35 | RCT | CABG (+) valve, Aortic | No | No | HES 130/0.4 6% 20 ml.kg‾¹ + acetated Ringers | LR 2000 | Heparin 5000 IU | 2000 | No | Yes | No | Fluid balance, fluid requirements, blood loss, PRBCs (no SD), platelets, platelet transfusion requirements (no SD) |
| Skhirtladze et al. 2014 | 240 | RCT | CABG (+) Valve, Aortic | No | No | 1: HES 130/0.4 6% (max 50 ml.kg‾¹.day‾¹) + LR  2: Alb 5% (max 50 ml.kg‾¹.day‾¹) + LR | LR 1500 | Mannitol 20% 100, heparin 5000 IU | 1600 | No | Yes | No | Fluid balance, fluid requirements (median + IQR), blood loss (median + IQR), PRBCs (median + IQR), platelets (median + IQR) |
| Svendsen et al. 2018 | 38 | RCT | CABG | Yes | No | HES 130/0.4 6% 1700 | Acetated Ringers 1700 | NR | 1700 | No | NR | NR | Fluid balance, blood loss |
| Tigchelaar et al. 1997 | 36 | RCT | CABG | No | No | 1: HES 10% 500 (max 2 mg.kg‾¹.24h) + LR 1500  2: Alb 20% 400 + LR  1600  3: gelatin 3% 2000 (max 3000ml.24h) | None | Heparin 1500 IU | 2000 | No | Yes | Yes | Blood loss (no data) |
| Tiryakioglu et al. 2008 | 140 | Prosp | CABG | NR | No | HES 130/0.4 6%  1500 | Ringers 1500 | Mannitol 200, NaHCO³‐ 60, heparin 150 IU.kg‾¹ | 1760 | No | NR | NR | Haematocrit, fluid balance, fluid requirements, blood loss, PRBCs (no SD), platelets, platelets transfusion requirements (no data) |
| Vanhoonacke et al. 2009 | 154 | RCT | CABG | No | No | 1: HES 130/0.4 6% 1500  2: modified fluid gelatin 1500 | None | NR | 1500 | No | Yes | Yes, post-operative HES in both groups | Haematocrit (no data), fluid requirements, blood loss, PRBCs, platelets, platelet transfusion requirements |
| Yanartas et al. 2015 | 132 | RCT | CABG | Yes | No | HES 130/0.4 6% 10 ml.kg‾¹ + Ringers 10 ml.kg‾¹ | Ringers 20 ml.kg‾¹ | Mannitol 20% 0.5g.kg‾¹, NaHCO³‐ 7.5% 1ml.kg‾¹, heparin 150 IU.kg‾¹ | 1500 | No | Yes | No | Haematocrit, fluid balance, fluid requirements (median + IQR), blood loss (median + IQR), PRBCs (median + IQR), platelets (median + IQR), platelet transfusion requirements (no data). |
| Zarro et al. 2001 | 53 | Prosp. | CABG | Yes | No | Alb 5% 250 + Isolyte S 2000 | Isolyte S 2200 | NR | 2250/2200 | No | NR | NR | Haematocrit, fluid balance, weight gain, PRBCs, platelet transfusion |

Summary of included studies. Abbreviations: Alb, albumin; CABG, coronary artery bypass grafting; COP, colloid oncotic pressure; HES, hydroxyethyl starch; IQR, interquartile range; LR, lactated ringers; PRBCs, packed red blood cells; SD, standard deviation; NR, not reported.
